# Metagenomic libraries data sets from the hypersaline benthic microbial mats of the Fraternidad Lagoon, Puerto Rico, using an indirect DNA extraction method

**DOI:** 10.1128/mra.01504-25

**Published:** 2026-02-27

**Authors:** Rene Nieves-Morales, Christian J. Quiles-Pérez, Edwin Omar Rivera-Lopez, Irimar Torres-Zapata, Josue Rodriguez-Ramos, Carlos Rios-Velazquez

**Affiliations:** 1 Microbial Biotechnology and Bioprospecting Laboratory, Biology Department, University of Puerto Rico, Mayagüez, Puerto Rico, USA; 2 Department of Microbiology, The Ohio State University 2647, Columbus, Ohio, USA; 3 Department of Food Science, The Pennsylvania State University 8082, University Park, Pennsylvania, USA; 4 Pacific Northwest National Laboratory, Biological Sciences Division, Richland, Washington, USA; Montana State University, Bozeman, Montana, USA

**Keywords:** microbial mats, hypersaline, metagenomics, metagenomic libraries

## Abstract

Microbial mats are biofilm formations that reflect early Earth ecosystems. To investigate their microbial diversity, an indirect DNA extraction method was applied to benthic ephemeral microbial mats from Fraternidad Saltern Lagoon during rainy and dry seasons. This approach yields high molecular DNA, suitable for metabolic and diversity analysis.

## ANNOUNCEMENT

Microbial mats are composed of diverse communities of microorganisms that may resemble Earth’s earliest life forms (1–4). It is theorized that these ecosystems played a role in Earth’s atmosphere oxygenation, leading to oxygen-dependent life (5, 6). Molecular techniques advancements have revealed niche differentiations across microbial mat layers and their microbial communities, leading to the discovery of microbes and metabolic functions (7, 8). To assess taxonomic and metabolic diversity, we employed an indirect DNA extraction method of the benthic ephemeral hypersaline microbial mats of the Fraternidad Saltern Lagoon (FSL) enhancing DNA molecular weight recovery, facilitating functional metagenomic, and metabolic analysis (9, 10).

Benthic ephemeral mat samples were collected from the FSL, Cabo Rojo, Puerto Rico (17.98˚N, 67.21˚W) during the dry and rainy seasons (11), transported at room temperature and stored at 4°C. High-molecular-weight DNA was extracted using a modified indirect extraction protocol (12–14). Following homogenization and washing, microbial cells were embedded in low-melting-point agarose into plugs, cooled on ice, and stored at 4°C prior to cell lysis by incubating in buffer (0.01 M Tris, 0.05 M NaCl, 0.2 M EDTA [pH 8.0], 1% sarkosyl, 1% sodium deoxycholate, and 1 mg/mL lysozyme) for 3 h at 37°C. Protein digestion was performed with proteinase K (1 mg/mL) in ESP buffer (1% sarkosyl, 0.5 M EDTA [pH 8.0]) at 55°C for 24 h, followed by treatment with PMSF (phenylmethylsulfonyl fluoride; 1 mM, 2 h, room temperature) to inhibit enzymatic activity. DNA fragments exceeding 20 kb molecular weight were recovered by electrophoresis and electroelution (13, 14). Metagenomic DNA was assessed with a NanoDrop (Thermo Scientific NanoDrop Products) for quality (15), purified DNA was ligated into the fosmid vector pCC1FOS and packaged into lambda phages using Epicentre MaxPlax system and transduced into EPI300-T1R cells (16). DNA was purified with the QIAGEN Plasmid Miniprep Kit, fragmented, adapter-ligated, diluted to 4.0 nM, and sequenced on the Illumina MiSeq platform (2 × 300 bp paired-end reads, 30 cycles) with MiSeq Reagent Kit v3 (https://www.mrdnalab.com/). Total clone count was 1,400 for the rainy season and 30,000 for the dry season. 2,807,462 sequences were generated: 1,803,636 from the dry season and 1,003,826 from the rainy season. Raw reads were filtered using BBMap (bbmap.sh) with default settings and ambig=all to remove reads mapping to reference contaminants (EPI300-T1R host genome and pCC1FOS plasmid) (17) (Table 1). Data were analyzed using the National Microbiome Data Collaborative (NMDC) standardized metagenomic workflow (18), which included quality control (rqcfilter2 on BBTools v38.94), assembly (MetaSPAdes v4.0.0), gene prediction (Prodigal), taxonomic classification (Centrifuge v1.0.4), and functionally classified based on the DRAM metabolism hierarchy model (19–27). The bioinformatic workflow was processed in October 2025.

**TABLE 1:** Summary of annotation, quality metrics, and coding DNA sequences (CDS) for metagenomic data sets of Fraternidad Lagoon metagenomic libraries during the dry and rainy seasons

Season	Category	Metric	Value	Method
Dry	Annotation	Number of CDS	1,443	Prodigal v2.6.3
Dry	Annotation	Total CDS length	4,807,285 bp	Prodigal v2.6.3
Dry	Annotation	Median CDS length	657 bp	Prodigal v2.6.3
Dry	Annotation	Average CDS length	799.183 bp	Prodigal v2.6.3
Dry	Annotation	Shortest CDS	75 bp	Prodigal v2.6.3
Dry	Annotation	Longest CDS	9,075 bp	Prodigal v2.6.3
Dry	Annotation	CDS length standard deviation	609.897 bp	Prodigal v2.6.3
Dry	Annotation	Predicted features	6,014	Prodigal v2.6.3
Dry	Quality	Coding density	89.42%	
Dry	Quality	Genes per Mb	1,167.23	
Dry	Quality	Sequences per Mb	253.02	
Dry	MAGs		0	
Dry	Contig N50	bp	83	
Rainy	Annotation	Number of CDS	13,827	Prodigal v2.6.3
Rainy	Annotation	Total CDS length	19,893,291 bp	Prodigal v2.6.3
Rainy	Annotation	Median CDS length	528 bp	Prodigal v2.6.3
Rainy	Annotation	Average CDS length	666.018 bp	Prodigal v2.6.3
Rainy	Annotation	Shortest CDS	75 bp	Prodigal v2.6.3
Rainy	Annotation	Longest CDS	13,632 bp	Prodigal v2.6.3
Rainy	Annotation	CDS length standard deviation	530.592 bp	Prodigal v2.6.3
Rainy	Annotation	Predicted features	29,869	Prodigal v2.6.3
Rainy	Quality	Coding density	89.71%	
Rainy	Quality	Genes per Mb	1,407.51	
Rainy	Quality	Sequences per Mb	591.7	
Rainy	MAGs		9	
Rainy	Contig N50	bp	1,635	

**Fig 1:**
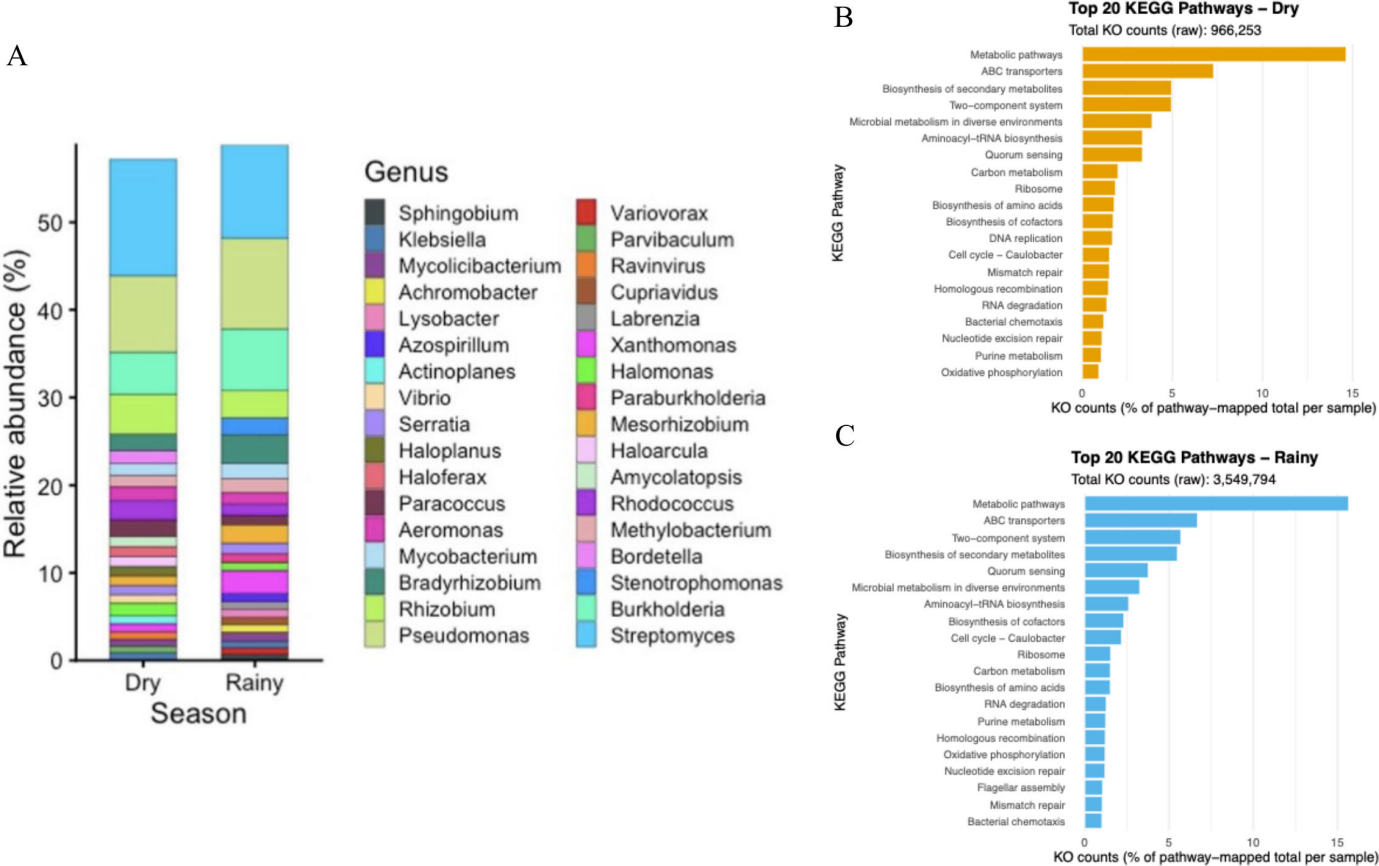
Taxonomic composition and functional profiles of benthic microbial mats from Fraternidad Saltern Lagoon across the dry and rainy seasons. (**A**) Relative abundance of the top 25 microbial genera of each benthic ephemeral microbial mat samples collected during the dry and rainy seasons. (**B** and **C**) Top 20 KEGG pathways identified from metagenomic data obtained during the dry (**B**) and rainy (**C**) seasons. Functional annotation was based on KEGG orthology (KO) assignments, and pathway abundance is expressed as percentages of the total counts.

Figure 1 analysis showed that both dry- and rainy-season mats were dominated by the genera *Streptomyces* (13.3% and 10.7%), *Pseudomonas* (8.7% and 10.3%), and *Burkholderia* (4.8% and 7.0%), respectively. Functional profiling indicated dry-season samples were enriched in carbon metabolism, amino acid biosynthesis, and membrane transport pathways. In contrast, rainy-season samples were characterized by cofactors biosynthesis, carbon metabolism, and amino acid biosynthesis. Although composition was similar, relative abundance between seasons' taxonomy and functional profiles might have been affected by weather changes.

## Data Availability

Under BioProject PRJNA1357061, sequence raw archives (SRA) are available under accessions SRS26982951 (Fraternidad Dry) and SRS26982952 (Fraternidad Rain). Corresponding assembled metagenomes are available under BioSample accessions SAMN53094954 (Dry Season) and SAMN53094955 (Rainy Season). Additionally, assembly, annotation, raw reads, and MAGs information (quality and sequences) are public in the Zenodo repository under the following link: https://doi.org/10.5281/zenodo.18644825.

## References

[1] Walter MR, Buick R, Dunlop JSR. 1980. Stromatolites 3,400–3,500 Myr old from the North Pole area, Western Australia. Nature 284:443–445. doi:10.1038/284443a0

[2] Allen MA, Goh F, Burns BP, Neilan BA. 2009. Bacterial, archaeal and eukaryotic diversity of smooth and pustular microbial mat communities in the hypersaline lagoon of Shark Bay. Geobiology 7:82–96. doi:10.1111/j.1472-4669.2008.00187.x 19200148

[3] Ruvindy R, White RA 3rd, Neilan BA, Burns BP. 2016. Unravelling core microbial metabolisms in the hypersaline microbial mats of Shark Bay using high-throughput metagenomics. ISME J 10:183–196. doi:10.1038/ismej.2015.87 26023869 PMC4681862

[4] Spring S, Brinkmann N, Murrja M, Spröer C, Reitner J, Klenk HP. 2015. High diversity of culturable prokaryotes in a lithifying hypersaline microbial mat. Geomicrobiol J 32:332–346. doi:10.1080/01490451.2014.913095

[5] Hoehler TM, Bebout BM, Des Marais DJ. 2001. The role of microbial mats in the production of reduced gases on the early Earth. Nature 412:324–327. doi:10.1038/35085554 11460161

[6] Des Marais DJ. 2003. Biogeochemistry of hypersaline microbial mats illustrates the dynamics of modern microbial ecosystems and the early evolution of the biosphere. Biol Bull 204:160–167. doi:10.2307/1543552 12700147

[7] Berg IA, Kockelkorn D, Buckel W, Fuchs G. 2007. A 3-hydroxypropionate/4-hydroxybutyrate autotrophic carbon dioxide assimilation pathway in archaea. Science 318:1782–1786. doi:10.1126/science.1149976 18079405

[8] Rinke C, Schwientek P, Sczyrba A, Ivanova NN, Anderson IJ, Cheng J-F, Darling A, Malfatti S, Swan BK, Gies EA, Dodsworth JA, Hedlund BP, Tsiamis G, Sievert SM, Liu W-T, Eisen JA, Hallam SJ, Kyrpides NC, Stepanauskas R, Rubin EM, Hugenholtz P, Woyke T. 2013. Insights into the phylogeny and coding potential of microbial dark matter. Nature 499:431–437. doi:10.1038/nature12352 23851394

[9] Berry AE, Chiocchini C, Selby T, Sosio M, Wellington EMH. 2003. Isolation of high molecular weight DNA from soil for cloning into BAC vectors. FEMS Microbiol Lett 223:15–20. doi:10.1016/S0378-1097(03)00248-9 12798994

[10] Lam KN, Cheng J, Engel K, Neufeld JD, Charles TC. 2015. Recursos actuales y futuros para la metagenómica funcional. Front Microbiol 6. doi:10.3389/fmicb.2015.01196 PMC4625089 26579102

[11] Torres-Zapata I. 2012. Generation of large insert metagenomic libraries from subtropical hypersaline microbial mats and their screening for antibiotic resistance. University of Puerto Rico Institutional Repository. Available from: https://hdl.handle.net/20.500.11801/655

[12] Casillas-Martinez L, Gonzalez ML, Fuentes-Figueroa Z, Castro CM, Nieves-Mendez D, Hernandez C, Ramirez W, Sytsma RE, Perez-Jimenez J, Visscher PT. 2005. Community structure, geochemical characteristics and mineralogy of a hypersaline microbial mat, cabo rojo, PR. Geomicrobiol J 22:269–281. doi:10.1080/01490450500182672

[13] Liles MR, Williamson LL, Rodbumrer J, Torsvik V, Goodman RM, Handelsman J. 2008. Recovery, purification, and cloning of high-molecular-weight DNA from soil microorganisms. Appl Environ Microbiol 74:3302–3305. doi:10.1128/AEM.02630-07 18359830 PMC2394920

[14] Torres-Zapata I, Gonzalez-Montalvo A, Castro-Ruiz C, Rios-Velazquez C. 2010. Generation of large insert metagenomic libraries using indirect DNA extraction methods from benthic and ephemeral tropical hypersaline microbial mats, p 1569–1575. *In* Méndez-Vilas A (ed), Current research, technology and education topics in applied microbiology and microbial biotechnology. Formatex Research Center.

[15] Desjardins P, Conklin D. 2010. NanoDrop microvolume quantitation of nucleic acids. J Vis Exp PMCID:2565. doi:10.3791/2565 PMC3346308 21189466

[16] Rodriguez-Reyes MG, Rios-Velazquez C. 2021. Stool microbiome dataset of the critically endangered Puerto Rican parrot (Amazona vittata). Data Brief 37:107175. doi:10.1016/j.dib.2021.107175 34169125 PMC8207182

[17] Bushnell Brian. 2014. BBMap: a fast, accurate. Splice-Aware Aligner.

[18] Eloe-Fadrosh EA, Ahmed F, Anubhav MB, Baumes J, Borkum M, Bramer L, Canon S, Christianson DS, Corilo YE, Davenport KW. 2022. The national microbiome data collaborative data portal: an integrated multi-omics microbiome data resource. Edited by Elisha M. Wood-Charlson, Yan Xu, Patrick S. G. Chain, Lee Ann McCue, Douglas Mans, Christopher J. Mungall, Nigel J. Mouncey, and Kjiersten Fagnan. Nucleic Acids Res 50:D828–D836. doi:10.1093/nar/gkab990

[19] Bushnell B. 2014. BBMap: a fast, accurate, splice-aware aligner, Lawrence Berkeley National Laboratory. https://escholarship.org/uc/item/1h3515gn.

[20] Nurk S, Meleshko D, Korobeynikov A, Pevzner PA. 2017. metaSPAdes: a new versatile metagenomic assembler. Genome Res 27:824–834. doi:10.1101/gr.213959.116 28298430 PMC5411777

[21] Chan PP. 2019. tRNAscan-SE: searching for tRNA genes in. Edited by M. Kollmar. Gene Prediction 1962:1–14. doi:10.1007/978-1-4939-9173-0_1 PMC6768409 31020551

[22] Griffiths-Jones S, Moxon S, Marshall M, Khanna A, Eddy SR, Bateman A. 2005. Rfam: annotating non-coding RNAs in complete genomes. Nucleic Acids Res 33:D121–4. doi:10.1093/nar/gki081 15608160 PMC540035

[23] Bland C, Ramsey TL, Sabree F, Lowe M, Brown K, Kyrpides NC, Hugenholtz P. 2007. CRISPR recognition tool (CRT): a tool for automatic detection of clustered regularly interspaced palindromic repeats. BMC Bioinformatics 8:209. doi:10.1186/1471-2105-8-209 17577412 PMC1924867

[24] Hyatt D, Chen G-L, Locascio PF, Land ML, Larimer FW, Hauser LJ. 2010. Prodigal: prokaryotic gene recognition and translation initiation site identification. BMC Bioinformatics 11:119. doi:10.1186/1471-2105-11-119 20211023 PMC2848648

[25] Besemer J, Lomsadze A, Borodovsky M. 2001. GeneMarkS: A self-training method for prediction of gene starts in microbial genomes. Nucleic Acids Res 29:2607–2618. doi:10.1093/nar/29.12.2607 11410670 PMC55746

[26] Kim D, Song L, Breitwieser FP, Salzberg SL. 2016. Centrifuge: rapid and sensitive classification of metagenomic sequences. Genome Res 26:1721–1729. doi:10.1101/gr.210641.116 27852649 PMC5131823

[27] Shaffer M, Borton MA, McGivern BB, Zayed AA, La Rosa SL, Solden LM, Liu P, Narrowe AB, Rodríguez-Ramos J, Bolduc B, Gazitúa MC, Daly RA, Smith GJ, Vik DR, Pope PB, Sullivan MB, Roux S, Wrighton KC. 2020. DRAM for distilling microbial metabolism to automate the curation of microbiome function. Nucleic Acids Res 48:8883–8900. doi:10.1093/nar/gkaa621 32766782 PMC7498326

